# 1-Benzyl-3-[(4-meth­oxy­phen­yl)imino]­indolin-2-one

**DOI:** 10.1107/S2414314623004182

**Published:** 2023-05-19

**Authors:** Omobola A. Odedokun, Adebomi A. Ikotun, Chijioke J. Ajaelu, Nattamai Bhuvanesh

**Affiliations:** a Industrial Chemistry, Bowen University, Iwo, Osun State, Nigeria; b Department of Chemistry, Federal College of Education (Special), Oyo, Oyo State, Nigeria; c Department of Chemistry, Texas A & M University, College Station, Texas, USA; Benemérita Universidad Autónoma de Puebla, México

**Keywords:** crystal structure, *N*-benzyl­isatin, *p*-arnisidine, Schiff base

## Abstract

The title compound, C_22_H_18_N_2_O_2_, is a Schiff base obtained by condensing *p*-arnisidine (4-meth­oxy­aniline) with *N*-benzyl­isatin (1-benzyl-1*H*-indole-2,3-dione), which crystallizes in the triclinic *P*  space group. The benzyl and phenyl rings subtend dihedral angles of 76.08 (7) and 60.70 (6)°, respectively, with the isatin group. The imino C=N double bond exists in an *E* conformation.

## Abstract 2

The title Schiff base was obtained from the reaction of *N*-benzyl­isatin with *p*-arnisidine. The imino C=N double bond, exists in an *E* conformation.

## Graphical abstract

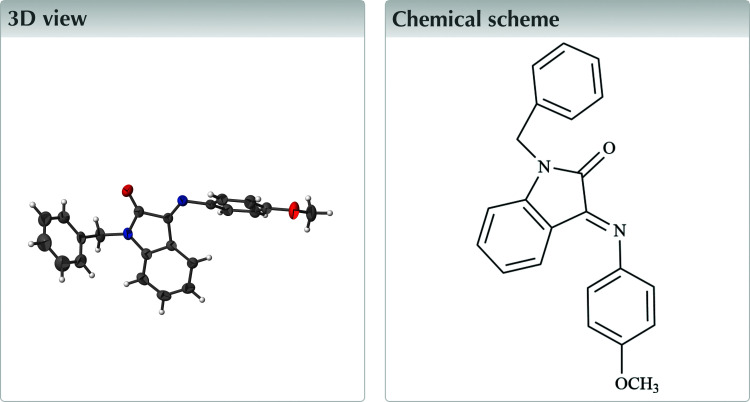

## Structure description

**Figure 1:**
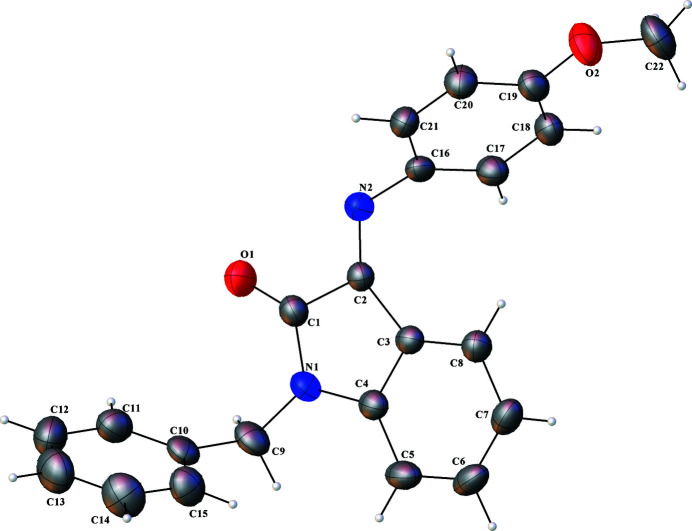
The mol­ecular structure of 1-benzyl-3-[(4-meth­oxy­phen­yl)imino]­indolin-2-one showing the atomic labelling; displacement ellipsoids are drawn at the 50% probability level.

Isatin (1*H*, indole-2,3-dione), an indole, and its analogs are an important class of heterocyclic compounds due to the presence of the indole ring structure, which is common to many pharmaceutical agents (Visagaperumal *et al.*, 2018 ▸). Isatin and its derivatives have served as starting materials for several organic, metal–organic and organometallic syntheses (Garima & Sumitra 2014 ▸; Ikotun *et al.*, 2015 ▸, 2019 ▸). These compounds attract great inter­est because of their potent pharmacological and biological activities (Guo, 2019 ▸; Czeleń *et al.*, 2022 ▸; Ikotun *et al.*, 2022 ▸). *N*-Benzyl­isatin is a bio­logically potent derivative of isatin that has been used to prepare many new biologically potent Schiff bases and complexes suitable for medicinal purposes (Shakir & Al-Mudhafar, 2020 ▸; Banerjee, 2021 ▸). The crystal structure of *N*-benzyl­isatin has been determined (Akkurt *et al.*, 2006 ▸; Schutte *et al.*, 2012 ▸). We have previously reported the synthesis and crystal structure of the Schiff base prepared from *N*-benzyl­isatin and *p*-toluidine (Ikotun *et al.*, 2012 ▸). The crystal structure of 1-benzyl-3-[(4-meth­oxy­phen­yl)imino]­indolin-2-one (Fig. 1 ▸) is hereby reported.

In the title compound, the asymmetric unit of compound contains one independent mol­ecule crystallizing in the triclinic space group *P* 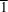 . The crystal disintegrated at 300 K and the X-ray structure was acquired at room temperature. The benzyl and phenyl rings subtend dihedral angles of 76.08 (7) and 60.70 (6)°, respectively, with the isatin group.

## Synthesis and crystallization

*N*-benzyl­isatin was prepared according to a literature method (Ikotun *et al.*, 2012 ▸). *N*-Benzyl­isatin (1.000 g, 4.2194 mmol) was dissolved in 20 ml of methanol. 4-Meth­oxy­laniline (0.5196 g, 4.2194 mmol) was also dissolved in 10 ml of methanol. The two solutions were mixed together while stirring at room temperature with the addition of 6 drops of glacial acetic acid for 8 h. The precipitate was filtered under vacuum, dried and the weight was determined to be 1.0566 g (73%). X-ray-suitable crystals were obtained by recrystallization from di­methyl­formamide solution after about two weeks.

## Refinement

Crystal data, data collection and structure refinement details are summarized in Table 1 ▸.

## Supplementary Material

Crystal structure: contains datablock(s) I. DOI: 10.1107/S2414314623004182/bh4074sup1.cif

Structure factors: contains datablock(s) I. DOI: 10.1107/S2414314623004182/bh4074Isup2.hkl

Supporting information file. DOI: 10.1107/S2414314623004182/bh4074Isup3.cml

CCDC reference: 2262787

Additional supporting information:  crystallographic information; 3D view; checkCIF report

## Figures and Tables

**Table 1:** Experimental details

Crystal data	
Chemical formula	C_22_H_18_N_2_O_2_
*M*_r_	342.38
Crystal system, space group	Triclinic, *P* 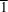
Temperature (K)	300
*a*, *b*, *c* (Å)	8.8872 (19), 8.8922 (19), 11.772 (3)
α, β, γ (°)	94.374 (6), 110.139 (6), 93.747 (6)
*V* (Å^3^)	866.7 (3)
*Z*	2
Radiation type	Mo *K*α
μ (mm^−1^)	0.09
Crystal size (mm)	0.55 × 0.53 × 0.44

Data collection	
Diffractometer	Bruker APEXII DUO (PHOTON 100)
Absorption correction	Multi-scan (*SADABS*; Krause *et al.*, 2015 ▸)
*T*_min_, *T*_max_	0.358, 0.431
No. of measured, independent and observed [*I* > 2σ(*I*)] reflections	24116, 4003, 3219
*R*_int_	0.049
(sin θ/λ)_max_ (Å^−1^)	0.651

Refinement	
*R*[*F* ^2^ > 2σ(*F* ^2^)], *wR*(*F* ^2^), *S*	0.044, 0.109, 1.03
No. of reflections	4003
No. of parameters	237
H-atom treatment	H-atom parameters constrained
Δρ_max_, Δρ_min_ (e Å^−3^)	0.28, −0.18

Computer programs: *APEX2* and *SAINT* (Bruker, 2018 ▸), *SHELXT* (Sheldrick, 2015*a* ▸), *SHELXL* (Sheldrick, 2015*b* ▸), *OLEX2* (Dolomanov *et al.*, 2009 ▸) and *PLATON* (Spek, 2020 ▸).

## full crystallographic data

### Crystal data

**Table:**

C_22_H_18_N_2_O_2_	*Z* = 2
*M**_r_* = 342.38	*F*(000) = 360
Triclinic, *P*1	*D*_x_ = 1.312 Mg m^−^^3^
*a* = 8.8872 (19) Å	Mo *K*α radiation, λ = 0.71073 Å
*b* = 8.8922 (19) Å	Cell parameters from 9907 reflections
*c* = 11.772 (3) Å	θ = 2.5–27.5°
α = 94.374 (6)°	µ = 0.09 mm^−^^1^
β = 110.139 (6)°	*T* = 300 K
γ = 93.747 (6)°	Block, orange
*V* = 866.7 (3) Å^3^	0.55 × 0.53 × 0.44 mm

### Data collection

**Table:**

Bruker APEXII DUO (PHOTON 100) diffractometer	3219 reflections with *I* > 2σ(*I*)
φ and ω scans	*R*_int_ = 0.049
Absorption correction: multi-scan (SADABS; Krause *et al.*, 2015)	θ_max_ = 27.5°, θ_min_ = 1.9°
*T*_min_ = 0.358, *T*_max_ = 0.431	*h* = −11→11
24116 measured reflections	*k* = −11→11
4003 independent reflections	*l* = −15→15

### Refinement

**Table:**

Refinement on *F*^2^	Hydrogen site location: inferred from neighbouring sites
Least-squares matrix: full	H-atom parameters constrained
*R*[*F*^2^ > 2σ(*F*^2^)] = 0.044	*w* = 1/[σ^2^(*F*_o_^2^) + (0.0428*P*)^2^ + 0.2626*P*] where *P* = (*F*_o_^2^ + 2*F*_c_^2^)/3
*wR*(*F*^2^) = 0.109	(Δ/σ)_max_ < 0.001
*S* = 1.02	Δρ_max_ = 0.28 e Å^−^^3^
4003 reflections	Δρ_min_ = −0.18 e Å^−^^3^
237 parameters	Extinction correction: *SHELXL2018/3* (Sheldrick 2015b), Fc^*^=kFc[1+0.001xFc^2^λ^3^/sin(2θ)]^-1/4^
0 restraints	Extinction coefficient: 0.184 (7)
Primary atom site location: dual	

### Special details

**Table:**

Geometry. All esds (except the esd in the dihedral angle between two l.s. planes) are estimated using the full covariance matrix. The cell esds are taken into account individually in the estimation of esds in distances, angles and torsion angles; correlations between esds in cell parameters are only used when they are defined by crystal symmetry. An approximate (isotropic) treatment of cell esds is used for estimating esds involving l.s. planes.
Refinement. Systematic reflection conditions and statistical tests of the data suggested the space group P-1. A solution was obtained readily using XT/XS in APEX2. Hydrogen atoms were placed in idealized positions and were set riding on the respective parent atoms. All non-hydrogen atoms were refined with anisotropic thermal parameters. Absence of additional symmetry and voids were confirmed using PLATON. The structure was refined (weighted least squares refinement on F2) to convergence (Sheldrick, 2008, 2015; Dolomanov, *et al.*, 2009).

### Fractional atomic coordinates and isotropic or equivalent isotropic displacement parameters (Å^2^)

**Table:**

	*x*	*y*	*z*	*U*_iso_*/*U*_eq_	
O1	0.32465 (13)	0.09548 (11)	0.17890 (9)	0.0467 (3)	
O2	0.15900 (14)	0.09727 (14)	0.84290 (10)	0.0546 (3)	
N1	0.33951 (14)	0.35590 (13)	0.20515 (10)	0.0367 (3)	
N2	0.28029 (13)	0.11348 (12)	0.40997 (10)	0.0340 (3)	
C1	0.32025 (15)	0.21291 (15)	0.23706 (12)	0.0336 (3)	
C2	0.28984 (14)	0.23196 (14)	0.35726 (11)	0.0290 (3)	
C3	0.29136 (14)	0.39654 (14)	0.38541 (11)	0.0295 (3)	
C4	0.32480 (15)	0.46621 (15)	0.29236 (12)	0.0324 (3)	
C5	0.34166 (17)	0.62236 (16)	0.29431 (14)	0.0429 (3)	
H5	0.363199	0.667768	0.232234	0.051*	
C6	0.32539 (17)	0.70910 (16)	0.39198 (15)	0.0455 (4)	
H6	0.335311	0.814188	0.394595	0.055*	
C7	0.29483 (17)	0.64298 (16)	0.48527 (14)	0.0429 (3)	
H7	0.286015	0.703664	0.550177	0.051*	
C8	0.27719 (16)	0.48616 (15)	0.48251 (12)	0.0355 (3)	
H8	0.256098	0.441593	0.545095	0.043*	
C9	0.37347 (17)	0.38347 (18)	0.09473 (13)	0.0426 (3)	
H9A	0.352646	0.486352	0.076706	0.051*	
H9B	0.300810	0.315234	0.027033	0.051*	
C10	0.54526 (17)	0.36126 (16)	0.10615 (12)	0.0364 (3)	
C11	0.5760 (2)	0.24368 (17)	0.03577 (14)	0.0458 (4)	
H11	0.490557	0.178863	−0.017936	0.055*	
C12	0.7322 (2)	0.2210 (2)	0.04411 (17)	0.0576 (4)	
H12	0.751328	0.142265	−0.004234	0.069*	
C13	0.8587 (2)	0.3160 (2)	0.12445 (18)	0.0634 (5)	
H13	0.963785	0.301025	0.130876	0.076*	
C14	0.8301 (2)	0.4332 (2)	0.19530 (17)	0.0612 (5)	
H14	0.916046	0.496349	0.250138	0.073*	
C15	0.67396 (19)	0.45734 (19)	0.18531 (14)	0.0484 (4)	
H15	0.655276	0.538342	0.231865	0.058*	
C16	0.24553 (15)	0.11827 (14)	0.51921 (11)	0.0311 (3)	
C17	0.10870 (16)	0.17623 (15)	0.52979 (12)	0.0360 (3)	
H17	0.039392	0.220029	0.465081	0.043*	
C18	0.07458 (16)	0.16936 (16)	0.63595 (13)	0.0372 (3)	
H18	−0.017670	0.207542	0.641944	0.045*	
C19	0.17839 (17)	0.10545 (15)	0.73296 (12)	0.0370 (3)	
C20	0.31359 (17)	0.04389 (16)	0.72204 (13)	0.0403 (3)	
H20	0.382694	−0.000043	0.786762	0.048*	
C21	0.34513 (16)	0.04796 (15)	0.61558 (12)	0.0360 (3)	
H21	0.433457	0.003537	0.607830	0.043*	
C22	0.0133 (2)	0.1394 (2)	0.85474 (16)	0.0595 (5)	
H22A	−0.076403	0.080458	0.793597	0.089*	
H22B	0.005243	0.245081	0.844710	0.089*	
H22C	0.012674	0.121264	0.933923	0.089*	

### Atomic displacement parameters (Å^2^)

**Table:**

	*U*^11^	*U*^22^	*U*^33^	*U*^12^	*U*^13^	*U*^23^
O1	0.0621 (7)	0.0413 (6)	0.0439 (6)	0.0032 (5)	0.0297 (5)	−0.0034 (5)
O2	0.0610 (7)	0.0759 (8)	0.0391 (6)	0.0185 (6)	0.0293 (5)	0.0136 (5)
N1	0.0435 (6)	0.0387 (6)	0.0353 (6)	0.0060 (5)	0.0217 (5)	0.0099 (5)
N2	0.0413 (6)	0.0296 (6)	0.0364 (6)	0.0042 (5)	0.0197 (5)	0.0051 (4)
C1	0.0334 (7)	0.0367 (7)	0.0335 (7)	0.0044 (5)	0.0151 (5)	0.0045 (5)
C2	0.0283 (6)	0.0302 (6)	0.0306 (6)	0.0040 (5)	0.0125 (5)	0.0035 (5)
C3	0.0267 (6)	0.0293 (6)	0.0335 (6)	0.0047 (5)	0.0109 (5)	0.0060 (5)
C4	0.0285 (6)	0.0342 (7)	0.0368 (7)	0.0063 (5)	0.0130 (5)	0.0084 (5)
C5	0.0419 (8)	0.0374 (8)	0.0553 (9)	0.0070 (6)	0.0212 (7)	0.0185 (7)
C6	0.0401 (8)	0.0275 (7)	0.0694 (10)	0.0055 (6)	0.0193 (7)	0.0067 (7)
C7	0.0409 (8)	0.0341 (7)	0.0533 (9)	0.0053 (6)	0.0175 (7)	−0.0032 (6)
C8	0.0370 (7)	0.0341 (7)	0.0374 (7)	0.0035 (5)	0.0159 (6)	0.0017 (5)
C9	0.0452 (8)	0.0564 (9)	0.0318 (7)	0.0070 (7)	0.0178 (6)	0.0154 (6)
C10	0.0448 (7)	0.0424 (8)	0.0281 (6)	0.0042 (6)	0.0188 (6)	0.0116 (5)
C11	0.0556 (9)	0.0420 (8)	0.0424 (8)	0.0003 (7)	0.0214 (7)	0.0037 (6)
C12	0.0699 (11)	0.0520 (10)	0.0632 (11)	0.0179 (8)	0.0368 (9)	0.0071 (8)
C13	0.0488 (9)	0.0777 (13)	0.0722 (12)	0.0166 (9)	0.0287 (9)	0.0157 (10)
C14	0.0473 (9)	0.0743 (12)	0.0554 (10)	−0.0031 (8)	0.0124 (8)	0.0016 (9)
C15	0.0524 (9)	0.0552 (9)	0.0375 (8)	0.0015 (7)	0.0179 (7)	−0.0024 (7)
C16	0.0382 (7)	0.0240 (6)	0.0341 (6)	0.0004 (5)	0.0172 (5)	0.0031 (5)
C17	0.0364 (7)	0.0363 (7)	0.0376 (7)	0.0058 (5)	0.0142 (6)	0.0096 (6)
C18	0.0329 (7)	0.0392 (7)	0.0442 (8)	0.0048 (5)	0.0193 (6)	0.0043 (6)
C19	0.0429 (7)	0.0380 (7)	0.0336 (7)	0.0012 (6)	0.0183 (6)	0.0027 (5)
C20	0.0449 (8)	0.0434 (8)	0.0349 (7)	0.0121 (6)	0.0143 (6)	0.0099 (6)
C21	0.0391 (7)	0.0323 (7)	0.0415 (7)	0.0082 (5)	0.0191 (6)	0.0055 (6)
C22	0.0575 (10)	0.0811 (13)	0.0523 (10)	0.0020 (9)	0.0361 (8)	0.0053 (9)

### Geometric parameters (Å, º)

**Table:**

O1—C1	1.2147 (16)	C10—C15	1.387 (2)
O2—C19	1.3697 (16)	C11—H11	0.9300
O2—C22	1.4197 (19)	C11—C12	1.387 (2)
N1—C1	1.3695 (17)	C12—H12	0.9300
N1—C4	1.4102 (17)	C12—C13	1.376 (3)
N1—C9	1.4663 (17)	C13—H13	0.9300
N2—C2	1.2753 (16)	C13—C14	1.376 (3)
N2—C16	1.4205 (16)	C14—H14	0.9300
C1—C2	1.5286 (17)	C14—C15	1.384 (2)
C2—C3	1.4739 (17)	C15—H15	0.9300
C3—C4	1.4056 (18)	C16—C17	1.3915 (18)
C3—C8	1.3895 (18)	C16—C21	1.3966 (19)
C4—C5	1.3843 (19)	C17—H17	0.9300
C5—H5	0.9300	C17—C18	1.3884 (19)
C5—C6	1.390 (2)	C18—H18	0.9300
C6—H6	0.9300	C18—C19	1.386 (2)
C6—C7	1.381 (2)	C19—C20	1.3934 (19)
C7—H7	0.9300	C20—H20	0.9300
C7—C8	1.3897 (19)	C20—C21	1.3776 (19)
C8—H8	0.9300	C21—H21	0.9300
C9—H9A	0.9700	C22—H22A	0.9600
C9—H9B	0.9700	C22—H22B	0.9600
C9—C10	1.513 (2)	C22—H22C	0.9600
C10—C11	1.384 (2)		
			
C19—O2—C22	118.34 (12)	C10—C11—C12	121.00 (15)
C1—N1—C4	110.94 (10)	C12—C11—H11	119.5
C1—N1—C9	122.28 (12)	C11—C12—H12	120.2
C4—N1—C9	126.77 (12)	C13—C12—C11	119.51 (16)
C2—N2—C16	122.11 (11)	C13—C12—H12	120.2
O1—C1—N1	125.81 (12)	C12—C13—H13	119.9
O1—C1—C2	127.73 (12)	C14—C13—C12	120.16 (16)
N1—C1—C2	106.44 (11)	C14—C13—H13	119.9
N2—C2—C1	117.81 (11)	C13—C14—H14	119.9
N2—C2—C3	136.54 (12)	C13—C14—C15	120.28 (16)
C3—C2—C1	105.47 (10)	C15—C14—H14	119.9
C4—C3—C2	106.74 (11)	C10—C15—H15	119.9
C8—C3—C2	133.75 (12)	C14—C15—C10	120.27 (15)
C8—C3—C4	119.38 (12)	C14—C15—H15	119.9
C3—C4—N1	110.38 (11)	C17—C16—N2	122.85 (12)
C5—C4—N1	128.15 (12)	C17—C16—C21	118.87 (12)
C5—C4—C3	121.46 (12)	C21—C16—N2	117.96 (11)
C4—C5—H5	121.1	C16—C17—H17	119.7
C4—C5—C6	117.89 (13)	C18—C17—C16	120.69 (12)
C6—C5—H5	121.1	C18—C17—H17	119.7
C5—C6—H6	119.2	C17—C18—H18	120.1
C7—C6—C5	121.59 (13)	C19—C18—C17	119.80 (12)
C7—C6—H6	119.2	C19—C18—H18	120.1
C6—C7—H7	119.9	O2—C19—C18	124.58 (12)
C6—C7—C8	120.26 (13)	O2—C19—C20	115.57 (12)
C8—C7—H7	119.9	C18—C19—C20	119.85 (12)
C3—C8—C7	119.41 (13)	C19—C20—H20	119.9
C3—C8—H8	120.3	C21—C20—C19	120.15 (13)
C7—C8—H8	120.3	C21—C20—H20	119.9
N1—C9—H9A	109.0	C16—C21—H21	119.7
N1—C9—H9B	109.0	C20—C21—C16	120.53 (12)
N1—C9—C10	112.92 (11)	C20—C21—H21	119.7
H9A—C9—H9B	107.8	O2—C22—H22A	109.5
C10—C9—H9A	109.0	O2—C22—H22B	109.5
C10—C9—H9B	109.0	O2—C22—H22C	109.5
C11—C10—C9	119.80 (13)	H22A—C22—H22B	109.5
C11—C10—C15	118.76 (14)	H22A—C22—H22C	109.5
C15—C10—C9	121.44 (13)	H22B—C22—H22C	109.5
C10—C11—H11	119.5		
			
O1—C1—C2—N2	6.2 (2)	C5—C6—C7—C8	−0.9 (2)
O1—C1—C2—C3	−177.95 (13)	C6—C7—C8—C3	0.3 (2)
O2—C19—C20—C21	−179.24 (13)	C8—C3—C4—N1	178.07 (11)
N1—C1—C2—N2	−174.78 (11)	C8—C3—C4—C5	−0.95 (19)
N1—C1—C2—C3	1.07 (13)	C9—N1—C1—O1	−1.9 (2)
N1—C4—C5—C6	−178.48 (13)	C9—N1—C1—C2	179.07 (11)
N1—C9—C10—C11	113.49 (15)	C9—N1—C4—C3	179.82 (12)
N1—C9—C10—C15	−67.07 (18)	C9—N1—C4—C5	−1.2 (2)
N2—C2—C3—C4	172.91 (14)	C9—C10—C11—C12	179.78 (14)
N2—C2—C3—C8	−2.5 (2)	C9—C10—C15—C14	179.05 (14)
N2—C16—C17—C18	175.73 (12)	C10—C11—C12—C13	0.6 (3)
N2—C16—C21—C20	−177.48 (12)	C11—C10—C15—C14	−1.5 (2)
C1—N1—C4—C3	−1.19 (15)	C11—C12—C13—C14	−0.4 (3)
C1—N1—C4—C5	177.75 (13)	C12—C13—C14—C15	−0.7 (3)
C1—N1—C9—C10	−74.72 (17)	C13—C14—C15—C10	1.7 (3)
C1—C2—C3—C4	−1.74 (13)	C15—C10—C11—C12	0.3 (2)
C1—C2—C3—C8	−177.20 (13)	C16—N2—C2—C1	−177.27 (11)
C2—N2—C16—C17	56.00 (18)	C16—N2—C2—C3	8.6 (2)
C2—N2—C16—C21	−130.53 (13)	C16—C17—C18—C19	0.6 (2)
C2—C3—C4—N1	1.84 (14)	C17—C16—C21—C20	−3.7 (2)
C2—C3—C4—C5	−177.19 (12)	C17—C18—C19—O2	177.83 (13)
C2—C3—C8—C7	175.61 (13)	C17—C18—C19—C20	−2.1 (2)
C3—C4—C5—C6	0.4 (2)	C18—C19—C20—C21	0.7 (2)
C4—N1—C1—O1	179.06 (13)	C19—C20—C21—C16	2.3 (2)
C4—N1—C1—C2	0.02 (14)	C21—C16—C17—C18	2.3 (2)
C4—N1—C9—C10	104.17 (15)	C22—O2—C19—C18	8.2 (2)
C4—C3—C8—C7	0.60 (19)	C22—O2—C19—C20	−171.81 (14)
C4—C5—C6—C7	0.6 (2)		
